# Isolation and characterization of Wharton’s jelly-derived multipotent mesenchymal stromal cells obtained from bovine umbilical cord and maintained in a defined serum-free three-dimensional system

**DOI:** 10.1186/1472-6750-12-18

**Published:** 2012-05-04

**Authors:** Tereza C Cardoso, Heitor F Ferrari, Andrea F Garcia, Juliana B Novais, Camila Silva-Frade, Marina C Ferrarezi, Alexandre L Andrade, Roberto Gameiro

**Affiliations:** 1 Laboratory of Animal Virology and Cell Culture, UNESP – University of São Paulo State, Araçatuba, São Paulo , 16050-680, Brazil; 2 Veterinary Hospital Surgery Service, Faculty of Veterinary Medicine, UNESP – University of São Paulo State, Araçatuba, São Paulo , 16050-680, Brazil

**Keywords:** Wharton´s jelly stem cells, Mesenchymal stromal cells, Differentiation, Immunomodulation

## Abstract

**Background:**

The possibility for isolating bovine mesenchymal multipotent cells (MSCs) from fetal adnexa is an interesting prospect because of the potential for these cells to be used for biotechnological applications. Bone marrow and adipose tissue are the most common sources of MSCs derived from adult animals. However, little knowledge exists about the characteristics of these progenitors cells in the bovine species. Traditionally most cell cultures are developed in two dimensional (2D) environments. In mammalian tissue, cells connect not only to each other, but also support structures called the extracellular matrix (ECM). The three-dimensional (3D) cultures may play a potential role in cell biotechnology, especially in tissue therapy. In this study, bovine-derived umbilical cord Wharton’s jelly (UC-WJ) cells were isolated, characterized and maintained under 3D-free serum condition as an alternative of stem cell source for future cell banking.

**Results:**

Bovine-derived UC-WJ cells, collected individually from 5 different umbilical cords sources, were successfully cultured under serum-free conditions and were capable to support 60 consecutive passages using commercial Stemline® mesenchymal stem cells expansion medium. Moreover, the UC-WJ cells were differentiated into osteocytes, chondrocytes, adipocytes and neural-like cells and cultured separately. Additionally, the genes that are considered important embryonic, POU5F1 and ITSN1, and mesenchymal cell markers, CD105^+^, CD29^+^, CD73^+^ and CD90^+^ in MSCs were also expressed in five bovine-derived UC-WJ cultures. Morphology of proliferating cells typically appeared fibroblast-like spindle shape presenting the same viability and number. These characteristics were not affected during passages. There were 60 chromosomes at the metaphase, with acrocentric morphology and intense telomerase activity. Moreover, the proliferative capacity of T cells in response to a mitogen stimulus was suppressed when bovine-derived UC-WJ cells was included in the culture which demonstrated the immunossupression profile typically observed among isolated mesenchymal cells from other species. After classified the UC-WJ cells as mesenchymal stromal phenotype the *in vitro* 3D cultures was performed using the AlgiMatrix® protocol. Based on the size of spheroids (283,07 μm ± 43,10 μm) we found that three weeks of culture was the best period to growth the UC-WJ cells on 3D dimension. The initial cell density was measured and the best value was 1.5 × 10^6^ cells/well.

**Conclusions:**

We described for the first time the isolation and characterization of UC-WJ cells in a serum-free condition and maintenance of primitive mesenchymal phenotype. The culture was stable under 60 consecutive passages with no genetic abnormalities and proliferating ratios. Taken together all results, it was possible to demonstrate an easy way to isolate and culture of bovine-derived UC-WJ cells under 2D and 3D serum-free condition, from fetal adnexa with a great potential in cell therapy and biotechnology.

## Background

Umbilical cord (UC) blood is currently used as a transplantable source of hematopoietic stem cells (HSCs) in humans, despite adult bone marrow representing the most common source of mesenchymal cells (MSCs) for clinical applications [1-4]. Recently, several groups have reported the isolation of adult SCs or mesenchymal stromal cells (MSCs) from human amniotic fluid, amniotic membrane and the umbilical cord [5]. Specifically, the MSCs have been isolated from umbilical cord, umbilical vein sub-endothelium, and the Wharton’s jelly (WJ) [6]. Within the WJ layer, MSCs have been isolated from three relatively indistinct regions: the perivascular zone, the intravascular zone, and the subamnion area [6]. However, whether MSCs isolated from the different compartments of the UC represent different populations remains unknown [6].

The WJ cells are not derived from UC but from the cushioning matrix in between the umbilical blood vessels [7,8]. This cell population meets the criteria for stem cells in that they can self-renew and can be induced to differentiate into various cell types [7]. Contrary to what has been observed for adult MSCs, WJ cells share several of the properties unique to fetal-derived MSCs. First, they have a greater expansion potential i*n vitro* than adult MSCs [8]. Second, WJ cells express HLA-class I surface markers but do not express HLA-class II markers [9]. Besides, UC-WJ cells sharing common surface markers with bone marrow MSCs, they also express low levels of transcription factors found in mouse and human embryonic stem cells [10]. These factors play a central role in the regulation of pluripotency and self-renewal. These factors include the POU (Pit/Oct/Unc) domain-containing protein Oct-4, Sox-2 and Nanog [9]. Indeed, it has been shown that WJCs are immune suppressive in mixed lymphocytes assays by inhibiting T-cell proliferation [2,7]. This is a desirable MSCs behavior to be use in cell therapy.

The MSCs population in Wharton´s jelly of UC has properties that make it of interest. For example, it is simple to harvest by non-invasive means, provides large number of cells without risk to the donor, could be expanded, genetically manipulated and differentiated *in vitro*[10-14]. Recently, MSCs were isolated from bovine umbilical cord blood for the first time [13], even though MSCs have been already isolated from sheep and horse [13,15-17]. The WJ-UC cell structure is embryonic in origin and it encloses the yolk sac, which is the source of primordial germ cells and the first hematopoietic stem cells [10].

Today, MSCs are usually expanded *in vitro* in culture media supplemented with fetal bovine serum (FBS), a highly variable and undefined component which is known to be an excellent additive for *in vitro* culturing of various cell types [18,19]. FBS is used in cell culture media as a supplement for robust undifferentiated MSC expansion, cell attachment, growth factors and vital nutrients. FBS contains xenogenic proteins, possibly inducing immunological responses and transmitting viral and prion diseases. It is also described the variability of lot-to-lot components [18,19].

The possibility of stem cell isolation for use in cell therapy motivates veterinary researchers to direct their studies towards new sources to obtain a relevant number of cells and to minimize risks for the donors and recipients [19]. In 2006, for the first time in veterinary medicine, Wharton’s jelly was obtained from porcine umbilical cord [15]. Because of the high potential of these cells to be subcultured and differentiated *in vitro*[18], they have generated great interest for their uses in cell and gene therapy, cloning, virological and biotechnological studies. In vitro stem cell research is commonly carried out by culturing cells as monolayers using conventional tissue culture techniques. Growing cells on plastic dishes provides a simplified and adequate approach to studying the effects of isolated niche components on stem cell activity. In an attempt to recreate this complex microenvironment and for more comprehensive understanding of the conditions experienced by cells in the body, the use of three-dimensional (3D) culture systems is gaining increasing attention [20]. Studies of the last years demonstrated that mimicking the components of the stem cell niche will facilitated self-renewal and controlled differentiation ex vivo [21,22]. Further, the establishment of a simplified stem cell niche *in vitro*, allows future works to study physiological as well as pathophysiological mechanisms on the stem cell.

The aim of this study was to isolate and propagate the bovine derived-UC-WJ cells collected from five pregnant calves by surgery procedure. Additionally, it was compared the characteristics in terms of growth kinetics, cell viability, telomerase activity, phenotype, plasticity and multipotency. This study highlights the possibility potential source of multipotent MSCs and may support many therapeutic and biotechnological roles in large animals.

## Results and discussion

### Isolation of bovine UC- WJ cells

**Figure 1:**
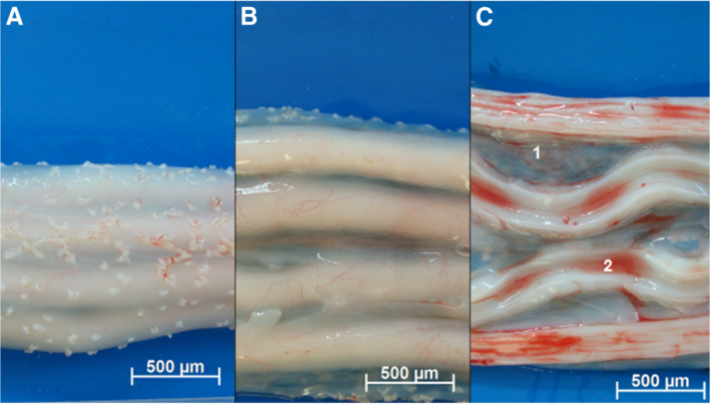
**Bovine umbilical cord.** ( **A**) Cut longitudinally with a scalpel ( **B**) and after removal of the Wharton’s jelly ( **C**) from the intervascular (1) and the discarded intravascular fluid (2). Bars 500 μm.

**Figure 2:**
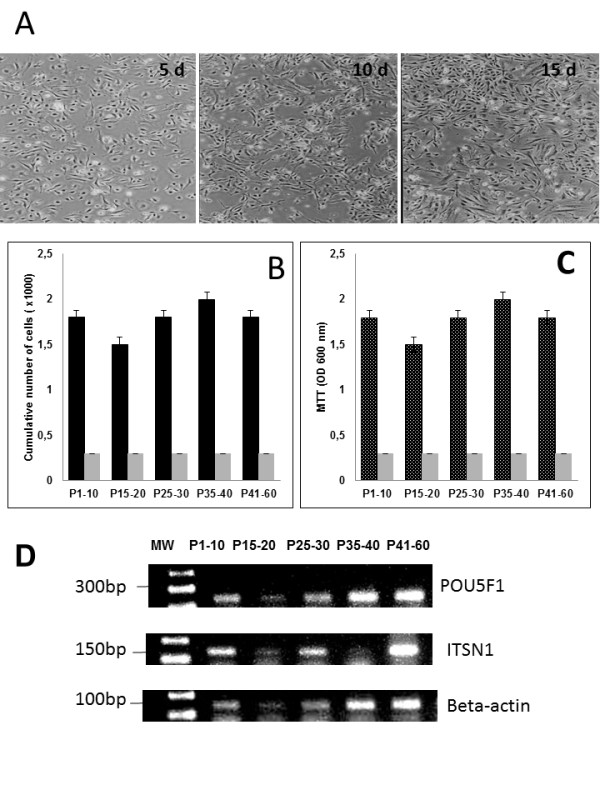
**Photomicrographs of undifferentiated and differentiated bovine UC-WJ cells in culture, cell concentration, viability, molecular characterization.** ( **A**) spindle-shaped fibroblast-like appearance can be observed under phase contrast microscopy; ( **B**) cell concentration (first column) and CFU (second column) expressed as number of cells per ml and number of colony forming units (CFU/ml); ( **C**) viability of bovine-derived UC-WJ cells measured by MTT based assay. Data are expressed as mean ± standard deviation (s.d.) of values; POU5F1 and ITSN1 expression in bovine-derived UC-WJ cells during cells passages. A typical profile of MSCs was exhibited at all passages, using the beta-actin gene as an internal control for reverse transcription-polymerase chain reaction. MW-molecular weight 1 kb plus.

The WJ collected from five bovine UC were isolated based on the capacity of MSCs to adhere to a plastic surface, without the need for any enzymatic digestion, stripping of the cord vessels or dissection (Figure 1A–C). The umbilical cords were sectioned into longitudinal segments (n = 5) to expose the WJ, and incisions were then made to the matrix with a sterile scalpel to expose a wider area of tissue for contact with the plastic surface (Figure 1C). The segments were plated, and colonies of cells were observed with fibroblastic morphology (Figure 2A). The cultures were continued until cells reached subconfluence (Figure 2A, third picture). The cells were then expanded by successive passages (P60) with no statistical differences among cultures. All five cultures were successful in producing viable cells at P60. The isolation of MSCs is based on the migratory and plastic adhesion capacities and normally show spindle shaped, plate adherent and able to be substantially subcultured *in vitro*. The bovine UC-WJ cells described in this study presented the same morphology for all 5 initial sources. To date, most groups have used an enzymatic treatment including several proteases, such as collagenase, hyaluronidase, or trypsin, which can be associated with or without a mechanical removal of the UC vessels [23-28]. However, it is well known that the drawback to this enzymatic digestion is the over digestion of tissue, which may result in diminished cellular viability, degradation of cellular surface receptors and altered cellular function [23]. The possibility of obtaining human MSCs from the umbilical cord matrix using nonenzymatic isolation has been recently established, however few reports describes this procedure for animal models [11,23]. This procedure diminishes the cell damage and increase cell viability which can explained the successful results obtained from five umbilical cords [11].

**Figure 3:**
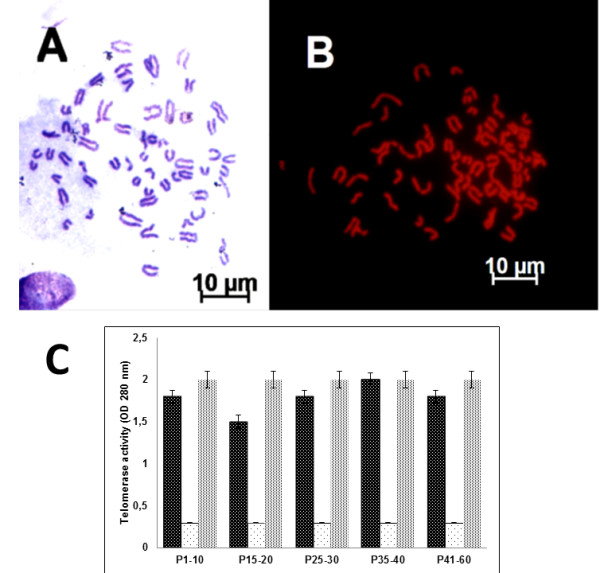
**Karyotype of bovine-derived UC-WJ cells.** ( **A**) Giemsa and ( **B**) propidium iodide (PI) staining. Bovine chromosomes at metaphase (2n = 60), reflecting acrocentric morphology. ( **C**) Telomerase repeat amplification results. The first bar represent the samples, second represents negative control and the third the positive control. Data are expressed as mean ± standard deviation (s.d.) of values.

Serum-free media has already been used for human MSC expansion and has been shown to result in better cell proliferation, as compared to medium containing FBS [18]. However, it has been demonstrated species-dependent differences when this procedure was applied in adipose derived stem cells with non-affected differentiation capacity [19]. In this study, it was possible to cultivate UC-WJ cells from five different sources using a serum-free condition and reach 60 consecutive passages with isolated bovine stromal MSCs.

### Cell viability, CFU, karyotype and telomerase activity analysis

After 15 days of culture, the first passage (P1) was performed, and from P1-10, P15-20, P25-30, P35-40 and P45-60 the MTT and colonies forming units CFU assays were applied. At P1-10, after 5 days of culture, 1.8 × 10^3^ cells / ml were obtained. From P1 to P60 the cell concentration remained between 1.5 - 2.0 × 10^3^ cells / ml (Figure 2B). The CFU was not altered from P1 to P60 with 800 CFUs counted on each plate (Figure 2B). To measure the viability of the bovine-derived UC-WJ cells in continuously cultured conditions, an MTT assay was applied. The only negative control that was used was the medium, and the results demonstrated that from P1 to P60, the cells were in good quality utilizing the culture system applied in this study (Figure 2C). Additionally, a positive correlation (*r* = 0.98) was found between cell viability and its concentration for five studied cultures (p < 0.05). To verify the cell viability, an MTT assay was applied, and the results revealed good *in vitro* adaptation of these cells using any growth factor in addition to medium. Moreover, the percentage of living cells did not decrease when the passage number remained at a constant level. Previous studies in humans as well as one study reported to date in dogs describe the same culture condition for UC-WJ cells, however the FBS was added as supplement [18,25]. Until now, this is the first report on bovine UC-WJ cells growth under serum-free medium.

The bovine UC-WJ cells had a normal karyotype after *in vitro* culture, which proves that the tissue culture procedure employed does not alter chromosomal organization (Figure 3A and B). In addition, the activity of telomerase was verified (Figure 3C). This enzyme represents a reverse transcriptase that can elongate telomeric repeats and usually is diminished after divisions, and this activity triggers cellular senescence and apoptosis. Besides, it has been described the importance of telomerase activity in MSCs [18,25]. Germ cells, stem cells, continuous cells and cancer cells express high levels of telomerase activity. This physiological mechanism maintains a stable telomere length with unlimited replication capacity revealed in MSCs isolated from dog foetal adnexa [25]. As expected, bovine UC-WJ cells expressed high levels of telomerase activity. To our knowledge, this is the first study to report that bovine UC-WJ cells show telomerase activity and this phenomenon could explain the long term of cell culture (n = 5) observe in this study.

### Differentiation of bovine-derived UC- WJ cells

Pluripotency was confirmed by the ability of bovine derived-UC-WJ cells to differentiate into osteocytes, chondrocytes, adipocytes and neuron-like cells for all 5 bovine UC-WJ cells. Importantly, this suggests that bovine derived-UC-WJ cells are capable of differentiation into multiple germ layers, an essential characteristic also observed in porcines, dogs, horses, duck and avian species [15-17,24-27,29-33].

To ensure that the MSCs properties were maintained during all passages, RT-PCR was used to measure mesenchymal and embryonic such as POU5F1 and ITSN1 gene expression. All undifferentiated bovine-derived UC-WJ cells expressed both genes in all passages (Figure 2D).

**Figure 4:**
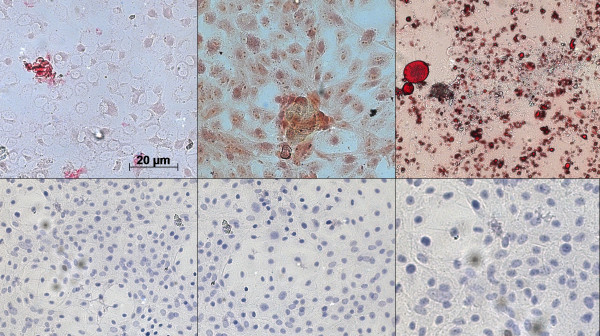
**Photomicrographs representative of the morphological appearance of chondrogenic, osteogenic and adipogenic differentiation of bovine-derived UC-WJ cells at P40.** For each differentiation protocol, undifferentiated cells were kept as controls ( **D, E, F**). **A**) Presence of calcium mineralization after 20 d of induction Alizarin Red staining; **B**) Presence of acidic proteoglycans were observed after 1 week of chondrogenic differentiation by Safranin O staining; **C**); adipogenic differentiation after 15 d showing lipid droplets stained with Oil Red (scale bar: 20 μm) ( **D**); after neurogenic induction of 2 week a neuronal-like morphology was visible under immunofluorescence microscopy positive N200 cell marker ( **E**) (scale bar: 20 μm).

Undifferentiated cells were included in all analysis (Figure 4A). To study chondrogenic differentiation, the cells were fixed and stained with Safranin O at 21 days of induction. The presence of glycosaminoglycans was shown (Figure 4B) as red deposits. Osteogenic differentiation was detected by the matrix calcification shown by Alizarin red staining. For all samples from P1 to P60, a mineralized matrix was formed after 20 days of induction (Figure 4C). The alkaline phosphatase activity for differentiated cells was intense (OD ≥0.9 measured at 405 nm) in contrast to undifferentiated cells (OD ≤0.1) (results not shown). This was observed at all passages. After induction, adipogenic differentiation of bovine-derived UC-WJ cells was observable between 10–15 days. The cells contained a high number of very small lipid vacuoles that stained positively using Oil Red solution (Figure 4D). The evidence of lipid vacuoles was frequently observed at P1-30, but less so from P30-40 (data not shown). The neurogenic induction occurred after 48 h when spindle-shaped cells began to contract and became to irregularly shaped (Figure 4E). After 1 week of induction, all UC-WJ cells in all passages expressed N200 (Figure 4E); however, the undifferentiated cells were negative for N200 (Figure 4F). Bovine derived- UC-WJ cells exhibited a typical MSC phenotype, with a gene expression profile that includes the expression of embryonic and mesenchymal cell markers at all cell passages. In mammals, embryonic markers are commonly expressed in pluripotent stem cells such as embryonic and induced MSCs and regulate self-renewal and pluripotency [9,15]. An important property to evaluate in stem cells to assess their usefulness in allogenic regenerative medicine is the expression of markers related to their immunogenicity [7,8]. The inhibition of T-cell proliferation in response to mitogens was observed. Future work includes studies to determine the capacity of bovine derived-UCWJ cells in regenerate damage tissues as already performed in horses [24,26].

### Expression of MSC markers on cell surface and suppression of T-cell proliferation

**Figure 5:**
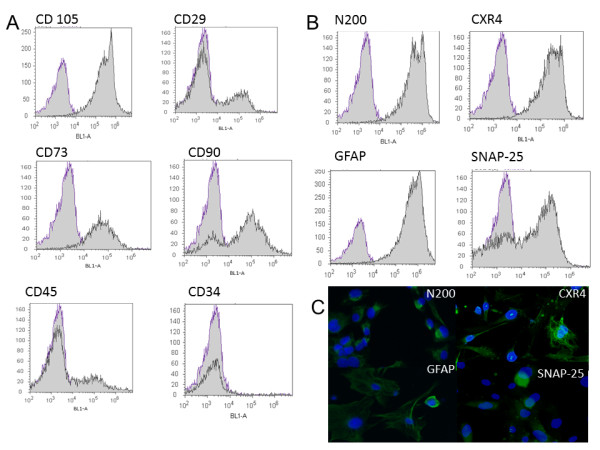
**Flow cytometric analyses of surface marker expression and neuronal induction in bovine-derived UC-WJ cells.A).**The data of negative isotype control and respective surface markers for mesenchymal stromal cells are shown by histogram. **B**) The data shown are the representative cell phenotype after neuronal induction. The data was processed by Attune™ acoustic focusing cytometer and excluded auto-fluorescence as a global compensation tool (≤ 10^3^). Y-axis represents log scale; **C**) After neurogenic induction of 2 week a neuronal-like morphology was visible under immunofluorescence microscopy positive N200, CXR4, GFAP and SNAP-25 cell markers (scale bar: 20 μm).

The density plot expressed the isotype negative control of flow cytometric analysis generates the histogram of negative control applied in all measures (Figure 5A and B). The expression patterns by immunophenotyping undifferentiated UC-WJ cells revealed cells positive for CD105, CD29, CD73 and CD90 MSCs markers (Figure 5 II C, G and H) and negative for CD45 and CD34. Both CD45 and CD34 are considered hematopoietic surface markers that were not expected to be expressed in UC-WJ cells as confirmed in the present study. To confirm the neurogenic potential of UC-WJ cells in this study, we measured the expression of CXCR4, SNAP-25, along with the neuronal cell markers N200 and GFAP. There were positive rates in N200 and CXR4, 70-90% (Figure 5 II A and B). However, fewer numbers of cells (20%) were positive for GFAP and SNAP-25 cell markers (Figure 5 II E and F). These results were repeated for all passages in this study. Last, we assessed the ability of bovine-derived UC-WJ cells to inhibit T-cell proliferation in response to mitogens. The addition of UC-WJ cells to blood monocytes stimulated with ConA or PMA/ionomycin inhibited their proliferation less than 20% in comparison to no addition of UC-WJ cells 89% of proliferation. This indicates that UC-WJs also exert immunosuppressive effects on bovine T-cells.

Contamination of UC-WJ cells with hematopoietic SCs is a concern when cells are isolated from tissues containing clotted blood [4,5,7,11]. Because bovine UC-WJ cells did not expressed both CD45^+^ and CD36^+^, which are considered to be a common leukocyte antigen and platelet collagen receptor, are expressed in hematopoietic progenitors, they may be all considered mesenchymal origin.

### UC-WJ in vitro 3D culture system

**Figure 6:**
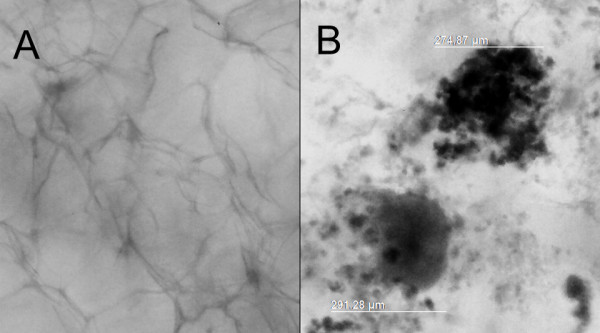
**UC-WJ cells cultured in 3D 6-well plates after 28 days post-seeding. A) **AlgiMatrix without cells considered as negative control; ** B) **1.5 × 10^6^ cells/well producing spheroids of 283, 07 μm (± 43,10 μm). Data are expressed as mean ± standard deviation (s.d.) of values.

Based on the size of spheroids recommended by manufacture´s (± 300 μm) we found out that 1 × 10^6^ cells/well and three week of observation was sufficient to produce 20 (± 4) spheroids/well with size of 284,12 μm (± 40,21) as shown in Figure 6B. This is the first report describing the UC-WJ derived from bovine cultured in 3D system. The advantage of AlgiMatrix® system is based mainly on biocompatibility and easy manipulation.

Significant alterations in cell behavior have been identified, when they are grown in 3D compared to 2D conditions; including changes in cell morphology, differentiation capacity, replicative ability, cell signaling, as well as significant increases in their therapeutic potential [21]. However, in veterinary stem cell therapy this condition has not been evaluated. The successful 3D culture in this study open new perspectives in MSCs studies involving animal model. However, further studies are still necessary to understand the mechanisms of cell-to-cell interactions, matrix remodeling and proliferation of animal MSCs cultured under 3D serum-free condition.

## Conclusions

This study provides a simplified isolation and characterization procedure for MSCs from Wharton’s jelly of the bovine umbilical cord and suggests that these cells have the potential to be a resource for isolating stem cells to be used for cloning, virological and biotechnological studies in veterinary medicine. Further studies are undergoing in order to establish a serum free and 3D culture media to support bovine-derived UC-WJ cells maintenance and evaluate their application on cell therapy and biotechnology.

## Methods

### Isolation of MSCs from WJ from bovine UC

Umbilical cords (UCs) were obtained from Nelore calves (n = 5) that had been reproduced by artificial insemination, kept in the same environment and delivered by cesarean section after a gestational age of 36–38 wk. Cesarean sections were performed because the cows presented dystocia by approaching from the flank ipsilateral to the pregnant corn. UCs were collected according to the Animal Care Committee at University of São Paulo State, Brazil, and they were conserved at room temperature in sterile phosphate buffered saline (PBS) supplemented with a penicillin/streptomycin solution containing penicillin 100 μg/ml, streptomycin 10 μg/ml and amphotericin B 250 μg/ml (Sigma-Aldrich, St Louis, MO, USA) until use (within 3 h). The umbilical cord segments (5–12 cm) were sectioned longitudinally to expose the WJ after 3 h (Figure 1A, B and C). Some incisions were made on the matrix with a sterile scalpel to expose a wider tissue area for plastic contact [6]. The cord sections were then transferred to 25 cm^2^ tissue culture flasks (*TPP*®, Zollstr, SW) and plated for 5 d in Stemline® Mesenchymal stem cell expansion medium (Sigma-Aldrich) and 2 mM L-glutamine (Glutamax®, Invitrogen®, Grand Island, NY, USA), and then they were incubated at 38.5°C in 5% CO_2_ in a humidified incubator. The medium was refreshed every 24 h, and pictures were taken to observe the morphology in five days interval (Figure 2A). The cells of passage (P) 1–10, P15-20, P25-30, P35-40 and P45-60 were harvested and replated on 24-well plates at the same cell density. Culture medium was changed every four days for cell growth dynamical analysis, and then growth curves of different passages were drawn.

The cells line (n = 5) were then expanded until they reached subconfluence (80–90%; Figure 2A, third picture), at which time they were harvested by detachment after a 5 min incubation period with 0.25% trypsin (Invitrogen®). They were then replated into culture flasks at a 1:5 split ratio. For passaging, 1 × 10^6^ cells were replated in 25 cm^2^ flasks in the same condition as in the culture, and the cells were analyzed for their capacity for viability, differentiation, phenotype, and karyotype as well as for culture behavior. Cells passaged until P40 were considered to be a lineage of bovine-derived UC-WJ cells.

### Colony-forming assay and viability MTT test

To obtain the number of bovine-derived UC-WJ progenitors, cells were evaluated from P1-10, P15-20, P25-30, P35-40 and P45-60 for their colony forming unit capacity. Briefly, 1 × 10^6^ cells were plated in a 10 cm^2^ Petri dish in the same medium plus 2 mM of L-glutamine; they were then incubated at 38.5°C in 5% CO_2_ in a humidified incubator. After 20 d the medium was discarded, and the adherent cells were stained with 1% crystal violet for 10 min. Plates were rinsed, and colonies of more than 50 cells were scored using an inverted microscope (Olympus IX 70, Tokyo, Japan).

Cell proliferation analysis was performed using the In Vitro Toxicology Assay® Kit, MTT-based assay (TOXI-1 Kit; Sigma-Aldrich®). After culturing for 3 d in 2 ml of culture medium (which is described previously), the MTT (tetrazolium salts) was measured following the manufacturer’s recommendations. Absorbance was measured at 600 nm with a Biophotometer (Eppendorf®, Hamburg, Germany). All reported values are means of triplicated samples.

### Reverse transcriptase polymerase chain reaction (RT-PCR)

Cell RNA was isolated from bovine-derived UC-WJ cells (P1-10, P15 -20, P25-30 and P35-40) using TRIzol (Invitrogen®). RNA concentrations were measured by absorbance at 260 nm with Biophotometer (Eppendorf™), and 2 μg total RNA was used for the reverse transcription reaction using Superscript II reverse transcriptase (Invitrogen®). The cDNA was amplified using Taq Platinum High Fidelity (Invitrogen®). The primers used were designed according to the GenBank data base (Table 1). The RNA templates were amplified at 33 to 45 cycles of 94°C (30 s), 58°C to 61°C (30 s), 72°C (1 min), followed by amplification at 72°C for 10 min. PCR products were visualized with ethidium bromide in a 12% agarose gel.

**Table 1:** Primers used for RT-PCR

Genes	Primer sequences (5` to 3`)	Size (bp)
Bov- beta actin	AGGAAGGAAGGCTGGAAGAG (forward) GAAATCGTCCGTGACATCAA (reverse)	126
POU5F1	ACCCAGGCTGATGTGGGGCTC (forward) TGTGGCTAATTTGCTGCAGGGTG (reverse)	300
ITSN1	ATGCAGTCAAGTTTACCACAG (forward) TGACTGAGGAACAGCCCACTCTGC (reverse)	128

### Karyotyping and telomerase activity

Karyotype analysis of bovine-derived UC-WJ cells at P1-10, P15-20, P25-30, P35-40 and P45-60 was performed according to the standard procedure [9]. Briefly, the cell line was processed with colcemid (Sigma-Aldrich®) at 1 μg/ml in culture medium for 2 h followed by three washes in a fixative (3 methanol:1 acetic acid). Cells that were under metaphase stage were submitted to KCL (3 M) and then spread onto slides for Giemsa and propidium iodide (PI) staining. Morphology was analyzed and chromosomes were counted and compared to the standard *Bos indicus* karyotype pattern.

A TRAPeze® Telomerase Detection Kit (Millipore™, CA, USA) was used to assess the telomerase activity for samples by measuring the capacity of the extracted enzyme to perform the amplification of the target telomeric repeat sequences (TS). This reaction was measured through a UV (280 nm) excitation filter in a Biophotometer (Eppendorf®). The positive control was made up of MDBK (Madin-Darby bovine kidney) cells maintained in our laboratory, and the negative control was the extract of the same cells that were incubated at 85°C for 10 min prior to inactivate the telomerase enzyme. The samples were considered positive when optical density (OD) was ≥ 0.2 and were negative when OD was ≤ 0.2.

### In vitro multilineage differentiation assay

The differentiation potential of bovine-derived UC-WJ cells was examined using cells at P1-10, P15-20, P25-30, P35-40 and P45-60. Adipogenic, ostegenic and chondrogenic differentiations were performed according to manufacturer’s instructions (STEMPRO® differentiation medium, Invitrogen®). The neurogenic differentiation was adapted from previously studies [4]. For the differentiation procedure cells were plated at 1 × 10^6^ cells / ml in 6-well culture plate (*TPP*®) under the same culture conditions described above. After seeding for 48 h, medium was replaced with osteogenic STEMPRO® medium (Invitrogen®) (2 ml / well), and every four days the medium was replaced with fresh medium. After 20 days of differentiation, the cells were fixed with 4% of paraformaldehyde (Sigma-Aldrich®), and calcium mineralization was assessed by Alizarin Red staining (Sigma-Aldrich®). In this procedure alkaline phosphatase activity was also measured by p-nitrophenyl phosphatase (p-NPP) substrate reactions using ALP™ assay reagents (Sigma-Aldrich®). In brief, cells were washed twice with 2 ml of PBS with 0.2% Triton X-100 by shaking for 20 min. Then, the cell layers were incubated with 500 μl of substrate (10 mM p-NPP, 1 mM MgCl_2_) for 30 min at 37°C. The reaction was stopped by adding 100 μl NaOH 1 mmol. The p-NNP formed was measured using a microplate reader with a 405 nm filter (Multiskan®, Labsystems, NY, USA).

For adipogenic differentiation, 1 × 10^6^ cells / ml were plated in the same way described for adipogenic differentiation. The cultured medium was changed to 2 ml of STEMPRO® adipogenic differentiation medium, which was replaced every 24 h. After 15 days of differentiation, the cells were fixed with 4% of paraformaldehyde and stained with Oil Red solution (0.3% of Oil Red powder dissolved in 60% of isopropanolol) for 10 min (Sigma-Aldrich®). Thereafter, cells were washed with 60% isopropanolol. The induction of adipogenic differentiation was apparent by intracellular the accumulation of lipid-rich vacuoles that were stained with Oil Red.

To induce chondrogenic differentiation, similar culture conditions were followed as described for adipogenic and osteogenic induction. However, 3 × 10^6^ cells / ml were seeded, and the STEMPRO® differentiation medium was supplemented with 100 μl of chondrogenic inducer (Invitrogen®). The cells were kept under this condition for one week, replacing medium every 24 h. The cells were fixed with 4% paraformaldehyde and stained with Safranin O to stain glycosaminoglycans (Sigma-Aldrich®).

The differentiation of bovine-derived UC-WJ cells into neural-like cells followed the procedure described above but with some modifications [34,35]. The cells were cultured as described above at a concentration of 3 × 10^6^ cells / ml. The induction was performed by replacing the cultured medium with Neurobasal^tm^ medium plus B-27 and neural supplement (Invitrogen®), 20 % FBS (Sigma-Aldrich®) and 3 μM of β-mercaptoethanol (Sigma-Aldrich®). The medium was replaced each 24 h over the course of 14 days.

### Immunocytochemistry

Cell staining was performed using the primary monoclonal antibodies described in Table 2. Cells were fixed with 4% of paraformaldehyde for 15 min after 48 h of culturing in Lab-Tek® chamber slides (Nunc^tm^, Rochester, NY, USA). The cells were permeabilized for 10 min at room temperature in 0.4% Triton X-100 diluted in PBS. The cells were incubated overnight with primary antibodies at 4°C. After three washes, the cells were incubated with secondary antibody rabbit anti-mouse FITC (Sigma-Aldrich®) at a 1:100 dilution. For nuclear staining, DAPI (1 mg / ml) was diluted in PBS and loaded onto samples for 15 min. The images were collected under an AxioImager® A.1 light and a ultraviolet microscope connected to AxioCam® MRc (Carl Zeiss, Oberkochen, Germany). Images were processed using AxioVision® 4.8 software (Carl Zeiss).

**Table 2:** Specifications of the primary antibodies used for flowcytometer (FC) and immunocytochemistry (IC)

	Antibody	Dilution		Species	Supplier
		**FC**	**IC**		
CD34	Hematopoetic percurssor cells and MSCs	1:50	1:100	Mouse	Sigma-Aldrich®
CD45	Anti-bone marrow lymphoid cells	1:50	1:100	Mouse	Sigma-Aldrich®
CD44	Cellular receptor for hyaluronic acid reactive with bone marrow nucleated cells	1:30	1:50	Mouse	Sigma-Aldrich®
CD90	Anti-THy1 antigen	1:30	1:50	Mouse	Sigma-Aldrich®
CD105	Anti-endoglin	1:50	1:25	Mouse	Sigma-Aldrich®
CD29	Anti-integrin β1	1:50	1:25	Mouse	Sigma-Aldrich®
CD73	Anti-nucleotidase	1:25	1:25	Mouse	Sigma-Aldrich®
Anti- Vimentin	Anti-vimentin clone V9	1:50	1:500	Mouse	Sigma-Aldrich®
Anti- cytokeratin	Anti-Pan cytokeratin clone PCK-26	1:100	1:100	Mouse	Sigma-Aldrich®
GFAP	Glial fribrillary acidic protein	1:50	1:500	Mouse	Dako®
CXR4	CXC chemokine receptor 4	1:50	1:150	Mouse	Calbiochem®
SNAP-25	Synaptosomal- associated 25 kDa protein	1:50	1:100	Mouse	Prestige Antibodies™
Anti-N200	Meurofilament 200 Kda	1:100	1:100	Mouse	Sigma-Aldrich®

### Flow cytometry

Next, 1 × 10^6^ cells were harvested after detachment with 0.25% trypsin (Sigma-Aldrich®), washed in PBS and incubated for 18 h at 4°C with the same monoclonal antibodies described for immunocytochemistry (Table 2). After incubation with primary antibodies, the cells were washed three times with PBS plus 0.1% Triton X-100. Next, a 1:50 dilution of the secondary antibody was added to100 μl of cell suspension, and was then incubated at 37°C for 30 min. The cell suspension was washed as previously described, and after the final wash, the cells were fixed with 4% paraformaldehyde. Data were acquired with the Attune^tm^ acoustic focusing cytometer system (Applied Biosystems®, Foster City, CA, USA). The negative pattern was examined by applying the same cell suspension with the first incubation, and the result was included on the global compensation to exclude the auto fluorescence. A BL1-A (488 nm) filter was used in each analysis.

### T-cell proliferation assay

Bovine blood was prepared by centrifugation over Ficoll-Hypaque gradient (Sigma-Aldrich®). The cells were suspended in triplicate in 100 μL RPMI 1640 medium supplemented with 5% FBS, 2 mM L-glutamine (Invitrogen®), 0,1 mg / ml streptomycin, and 100 U/ml penicillin G (Invitrogen®) in 96-well plate in the presence or absence of bovive derived UC-WJ cells. The cultures were stimulated or unstimulated with mitogen (ConA or Phorbol 12-myristate 13-acetate (PMA) and ionomycin) and incubated for 48 h at 38.5°C. The cells were pulsed with [^3^ H] thymidine during the last 5 h of incubation. Lymphoproliferation was measured as counts per minute by a Matrix9600 beta counter (Packard Instrument Co., Meridien, CT). All experiments were performed in triplicate. The ConA was used at 5 μg/ml, PMA and ionomycin at concentration of 50 ng/ml and 1 μg/ml, respectively (Sigma-Aldrich®).

### Development and optimization of in vitro 3D culture model

It was performed the 3D culture of UC-WJ cells in a 6-weel culture plates at three different concentrations 0.5 × 10^6^, 1 × 10^6^ and 1.5 × 10^6^ cells/well in order to determine the optimal incubation density. The 3D cultures were daily observed during 28 days. Brief, 2 ml of UC-WJ cells suspensions containing firming buffer was added to the AlgiMatrix® (Invitrogen) 3D 6- well culture plates. After 5 min, 5 ml of Stemline® mesenchymal stem cell expansion medium was added to each well and incubated at 38.5 °C at 5% of CO_2_ in a humidified incubator. A fresh medium was added daily. The growth of cell spheroids was assessed by observing the respective size for each triplicate of cell suspension. The pictures were taken using an inverted microscope IX 70 (Olympus, Tokyo, Japan) and the size and number of spheroids measured using AxioVision® 4.8 software (Carl Zeiss).

### Statistical analysis

All statistical analyses were performed using SAS 9.1.2 software package (SAS Institute, Inc.). Data are presented as mean ± SD. Three replicates for each experiment were performed and the results represent these replicates. One-way analysis of variance (ANOVA) for multiple comparisons or two tailed student *t*-test, whenever applicable, was used. A level of P <0.05 was accepted as significant.

## Authors´ contributions

TCC, HFF and AFG participated in the design of the study, performed the cell culture in all steps, flow cytometric analysis and 3D culture. JBN, CSF and MCF participated the cow surgery and preparation of respective umbilical cords. ALA and RG participated also in the design of the study, performed the statistical analysis and drafted the manuscript. All authors read and approved the final manuscript.
